# Association between circulating inflammatory proteins and temporomandibular disorders: insight from a two-sample Mendelian randomization analysis

**DOI:** 10.1590/1678-7757-2024-0112

**Published:** 2025-01-10

**Authors:** Ao DING, Chan-Yuan YU, Feng JIANG, Chu-Yan WU, Jun ZHAO

**Affiliations:** 1 University of Science and Technology of China The First Affiliated Hospital of USTC Department of Stomatology Hefei China University of Science and Technology of China, The First Affiliated Hospital of USTC (Anhui Provincial Hospital), Department of Stomatology, Hefei, China.; 2 Obstetrics and Gynecology Hospital of Fudan University Department of Neonatology Shanghai China Obstetrics and Gynecology Hospital of Fudan University, Department of Neonatology, Shanghai, China.; 3 The First Affiliated Hospital of Nanjing Medical University Department of Rehabilitation Medicine Nanjing China The First Affiliated Hospital of Nanjing Medical University, Department of Rehabilitation Medicine, Nanjing, China.; 4 Shanghai Jiao Tong University School of Medicine College of Stomatology Shanghai Ninth People’s Hospital Shanghai China Shanghai Jiao Tong University School of Medicine, College of Stomatology, Shanghai Ninth People’s Hospital, Department of Orthodontics, Shanghai, China.; 5 Shanghai Jiao Tong University School of Medicine National Center for Stomatology Shanghai China Shanghai Jiao Tong University School of Medicine, National Center for Stomatology, Shanghai, China.; 6 National Clinical Research Center for Oral Diseases Shanghai China National Clinical Research Center for Oral Diseases, Shanghai, China.; 7 Shanghai Research Institute of Stomatology Shanghai Key Laboratory of Stomatology Shanghai China Shanghai Research Institute of Stomatology, Shanghai Key Laboratory of Stomatology, Shanghai, China.

**Keywords:** Mendelian randomization, Circulating inflammatory proteins, Temporomandibular disorders, Genome-wide association studies

## Abstract

**Background:**

Past studies have indicated links between specific inflammatory proteins in the bloodstream and temporomandibular disorders (TMDs). Nonetheless, there remains the need for further solid research pinpointing the exact causes behind these associations. This Mendelian randomization (MR) study aims to examine the association between 91 circulating inflammatory proteins and TMDs.

**Methodology:**

The most comprehensive genome-wide association studies available for circulating inflammatory proteins and TMDs was used in this two-sample MR analysis. The association between genetic predispositions to TMDs and levels of circulating inflammatory proteins was explored by various methods, including inverse variance weighted, MR-Egger, weighted median, simple mode, weighted mode, and MR-PRESSO techniques. To evaluate the reliability of these findings, sensitivity analyses such as Cochran’s Q test, the MR-Egger intercept test, and a leave-one-out approach were conducted.

**Results:**

Findings indicated significant links between lower levels of circulating CCL4 (odds ratio, OR: 0.9241, 95% confidence interval, CI: 0.8679-0.984, p=0.0138), IL-20 (OR: 0.8615, 95%CI: 0.7566-0.9808, p=0.0243), and TWEAK (OR: 0.8702, 95%CI: 0.7634-0.992, p=0.0375) and an increased risk of TMDs, according to the inverse variance weighted method. Conversely, a higher level of S100A12 in the blood stream was associated with an increased risk of TMDs (OR: 1.1368, 95%CI: 1.0134-1.2752, p=0.0286). Sensitivity analyses confirmed the stability of these outcomes.

**Conclusion:**

This study suggests that reduced levels of CCL4, IL-20, and TWEAK are associated with a higher risk of TMDs, alongside an increased risk of TMDs connected to elevated levels of S100A12.

## Introduction

Temporomandibular disorders (TMDs) manifest as discomfort in the jaw joint and surrounding muscles, sounds from the joint during movement, and either limited or abnormal movement of the jaw. These disorders significantly affect the quality of life for many and represent a considerable economic burden globally.^1^^,^^2^ Epidemiological research indicates that TMDs occur in approximately 5 to 12% of the population, ranking them as the second most frequent cause of chronic pain in the facial and oral regions.^3^ The Diagnostic Criteria for TMD^4^ classify them into two main categories: pain-related disorders and intra-articular TMDs. Pain-related disorders encompass conditions such as myalgia, local myalgia, myofascial pain, myofascial pain with referral, arthralgia, and headaches attributed to TMDs. On the other hand, intra-articular TMDs include disc displacement with reduction, disc displacement with intermittent locking, disc displacement without reduction with limited opening, disc displacement without reduction and without limited opening, degenerative joint disease, and subluxation. Currently, there exists no highly effective treatments for TMDs. Conservative treatment options for TMDs include medication, physiotherapy, occlusal splints, self-management strategies, and cognitive behavioral interventions.^5^^-^^10^

The multifactorial etiology of TMDs involves biological, environmental, social, emotional, and cognitive factors, all of which interact with one another.^11^^-^^14^ Inflammation is a critical factor in TMD development, with various inflammatory proteins, including cytokines, playing distinct roles in different TMD subtypes. Inflammatory proteins are molecules involved in immune responses and inflammation, such as cytokines, chemokines, and other signaling proteins. Pin-related TMDs, such as myalgia, show elevated levels of pro-inflammatory cytokines such as IL-6, IL-7, and IL-8.^15^ These cytokines contribute to pain and hyperalgesia by sensitizing nociceptors and triggering the release of additional inflammatory mediators, which have been linked to increased pain intensity and prolonged pain duration in TMD patients. In intra-articular TMDs, cytokines such as IL-1β and TNF-α promote joint tissue degeneration and remodeling by inducing the production of matrix metalloproteinases, which degrade extracellular matrix components, such as collagen and proteoglycans, resulting in joint dysfunction.^16^ IL-6 also contributes to cartilage degradation and inflammation within the joint, leading to bone and cartilage deterioration.^17^ Other inflammatory mediators, such as IFN-γ, PGE2, MMP-2, aggrecanase-1, and aggrecanase-2, may also contribute to these process.^18^^-^^20^ However, existing studies often involve small sample sizes and struggle to control confounding factors, limiting our understanding of the specific roles of circulating inflammatory proteins in TMDs.

Mendelian randomization (MR) offers a powerful epidemiological method that uses genetic variations, often single nucleotide polymorphisms (SNPs), as instrumental variables (IVs) to infer the causal influence of an exposure factor on a specific outcome. This technique is particularly valuable in situations in which randomized controlled trials are impractical or unethical, providing a robust alternative for estimating causal relationships with reduced bias. The random allocation of genetic variants at conception mimics the randomization process of randomized controlled trials, thereby minimizing the impact of confounding factors and reverse causality that commonly affect observational studies.^21^^,^^22^ MR has become increasingly popular for exploring causal links due to its ability to leverage extensive genetic data from genome-wide association studies (GWAS) and meta-analyses. However, the method also has limitations, including the requirement for strong genetic instruments, potential pleiotropy (in which genetic variants affect multiple traits), and challenges interpreting results. Despite these limitations, MR remains a valuable approach for investigating causal pathways in complex diseases. This study applied a two-sample MR approach to investigate the potential causal relationship between circulating inflammatory proteins and TMDs, providing new insights that could inform prevention and treatment strategies.

## Methodology

### Study design

**Figure 1:**
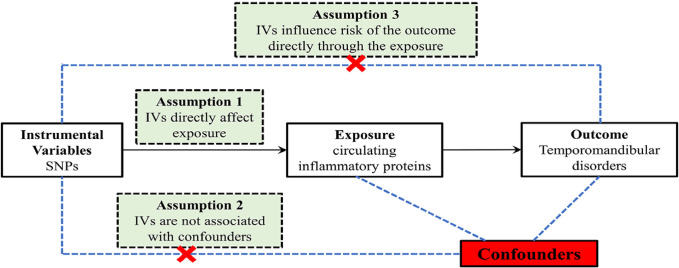
An overview of the study design. SNP: single nucleotide polymorphisms; IVs: instrumental variables.

This research aims to scrutinize the possible association between 91 circulating inflammatory proteins and TMDs by a two-sample MR methodology. Figure 1 succinctly illustrates the bidirectional MR study design in this investigation. The MR analysis adheres to three fundamental principles: relevance, indicating that the chosen genetic instruments are significantly correlated with the exposure variables; independence, ensuring that the genetic variations have no link with any confounding variable; and exclusion restriction, asserting that the genetic variations influence the outcomes solely by their effect on the exposure variables without any alternative routes.

### Data source

This research was conducted using de-identified, publicly accessible summary data from earlier GWAS, thereby eliminating the need for ethical approval. Specifically, for circulating inflammatory proteins, the analysis incorporates the most extensive GWAS available, which profiles genetic associations with 91 circulating inflammatory proteins across 14,736 individuals of European descent. The details of these inflammatory proteins are detailed in Table S1. The methodology to measure these proteins is thoroughly described in the original publication. For TMDs, GWAS summary statistics were sourced from the FinnGen consortium, specifically from its ninth release. The FinnGen project represents a comprehensive GWAS meta-analysis encompassing data from nine biobanks throughout Finland. With less than 3.3% sample overlap between the GWAS for circulating inflammatory proteins and that for TMDs, the potential for bias due to sample overlap is considered negligible. The FinnGen project has provided us with the most up-to-date dataset on TMDs. TMD cases were defined using the revised International Classification of Diseases version 10, specifically under code K07.6. This code encompasses diagnoses such as “snapping jaw,” “temporomandibular joint derangement,” “Costen complex or syndrome,” and “temporomandibular joint-pain-dysfunction syndrome.” Our study utilized a dataset from the FinnGen database, which includes 5,668 TMD cases and 205,355 controls diagnosed based on these criteria. Furthermore, our regression model incorporated covariates such as age, the top 10 principal components, and genotyping batch to ensure robust results. Detailed information of TMD dataset and diagnostic criteria are detailed in Table S2. Official endpoint definition website can be found at: https://r9.risteys.finngen.fi/endpoints/TEMPOROMANDIB. All involved original studies received ethical approval and were conducted with informed consent from participants.

### Instrumental variables

To adhere to the prerequisites of the MR methodology, it was imperative to select SNPs as IVs that showed significant correlations with the exposures of interest. A genome-wide significance level of p < 5×10^-8^ was used to pinpoint SNPs that were strongly associated with both TMDs and circulating inflammatory proteins. However, for some circulating inflammatory proteins, the number of SNPs meeting this stringent criterion was low. Consequently, a less strict significance threshold of 5×10^-6^ was chosen to include a broader set of SNPs associated with these proteins. To mitigate bias introduced by linkage disequilibrium, SNP clumping was performed with a distance parameter of 10,000 kilobases and a 0.001 r^2^ threshold. Additionally, palindrome SNPs were excluded from analysis. The robustness of each SNP as an instrumental variable was evaluated using the F-statistic, deeming SNPs with F-statistics greater than 10 as sufficiently strong to validate their use as instrumental variables for the analysis.

### Statistical analysis

The inverse-variance weighting (IVW) method was selected as the principal analytical approach in this study due to its superior statistical power and efficacy in scenarios devoid of pleiotropy among the instrumental variables.^23^ IVW combines genetic variant estimates to provide an overall causal effect, minimizing variance. To ensure a thorough evaluation of the causal links between circulating inflammatory proteins and TMDs, four additional MR methods were utilized: MR-Egger, weighted median, simple mode, and weighted mode. These techniques foster various forms of genetic pleiotropy, operating under different theoretical premises, thereby facilitating an examination of result consistency. Specifically, the weighted median method shows great resilience, offering dependable causal inferences even under the valid influence of most of its instrumental variables. Moreover, the MR-Egger method can consistently estimate causal effects even in instances in which no instrumental variables fully meet the MR assumptions, thus ensuring the robustness of our findings.

### F-statistic

For IVs to be considered valid in MR analysis, they must show a significant link with exposure. The F-statistic is a measure frequently used to gauge the strength of this association. The formula for calculating the F-statistic is: $F=R^{2}(n-k-1)/k\left(1-R^{2}\right)$ ，in which R^2^ represents the proportion of variance in the exposure that is explained by the genetic instrument, n is the total sample size, and k is the number of IVs being utilized. An F-statistic value below 10 suggests that the relationship between the IVs and the exposure is weak, leading to the exclusion of such IVs from further analysis to ensure the reliability and validity of the MR study findings.

### Sensitivity analysis

Several sensitivity analyses were conducted to ensure the integrity of this study and address potential MR assumption violations. The Cochran’s Q test gauged SNP heterogeneity in the IVW and MR-Egger methods. MR-PRESSO identified outliers. MR-PRESSO detected and corrected for horizontal pleiotropy in MR analyses, enhancing the reliability of causal inferences. A “leave-one-out” analysis checked the impact of individual SNPs on causal effect estimates, highlighting potential biases. The MR-Egger intercept test scrutinized pleiotropic links between genetic variations and confounders, with intercept deviations from zero indicating horizontal pleiotropy. These procedures utilized the “TwoSampleMR” package in R, version 4.3.2.

## Results

### Selection of instrumental variables

Adhering to the criteria for selecting instrumental variables, a total of 91 circulating inflammatory proteins encompassing 2154 SNPs (P < 1×10^-6^) served as IVs for the MR analysis. Table S3 thoroughly outline the specifics of all these SNPs.

### MR analysis

**Figure 2:**
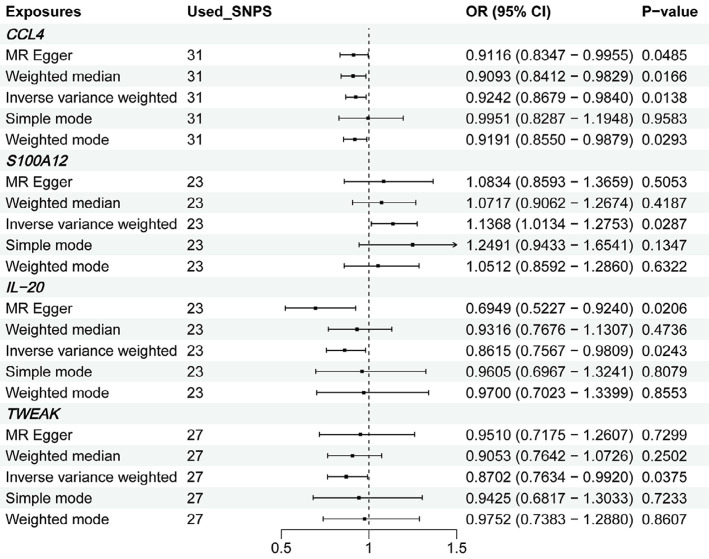
MR results of four circulating inflammatory proteins on TMDs.

Selecting IVW as the main method for MR analysis due to its superior statistical power, our study showed associations between four specific circulating inflammatory proteins and the risk of TMDs. We observed suggestive relationships indicating that genetically predicted decreases in the levels of CCL4 (OR: 0.9241, 95% CI: 0.8679-0.984, p=0.0138), IL-20 (OR: 0.8615, 95% CI: 0.7566-0.9808, p=0.0243), and TWEAK (OR: 0.8702, 95% CI: 0.7634-0.992, p=0.0375) are associated with a higher risk of developing TMDs. Conversely, an increase in S100A12 levels elevates TMDs risk (OR: 1.1368, 95% CI: 1.0134-1.2752, p=0.0286). The findings from the analysis of CCL4, IL-20, TWEAK, and S100A12 estimate magnitude and direction associations with those derived using the IVW method (Figure 2-4). The magnitude of these OR, which are close to 1, suggests moderate but potentially clinically significant effects, highlighting the importance of inflammatory pathways in TMD pathogenesis. These findings underscore the need for further investigating the modulation of these specific proteins as potential therapeutic targets.

**Figure 3 f03:**
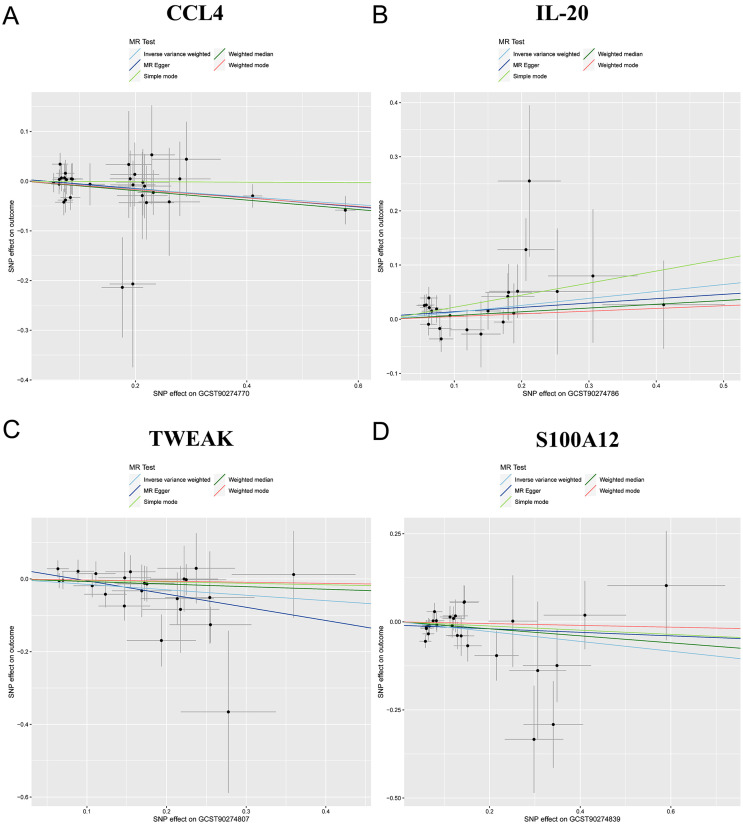
Scatter plots illustrating the genetic associations with four circulating inflammatory proteins. (A) CCL4, (B) IL-20, (C) TWEAK, (D) S100A12.

**Figure 4:**
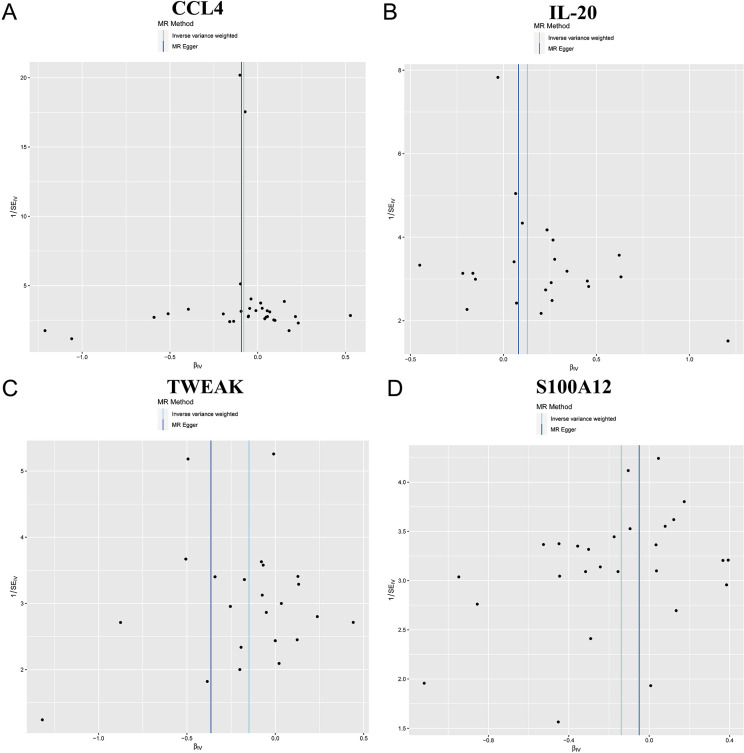
Funnel plot analyses of MR analyses for (A) CCL4, (B) IL-20, (C) TWEAK, and (D) S100A12.

### Sensitivity analysis

**Figure 5:**
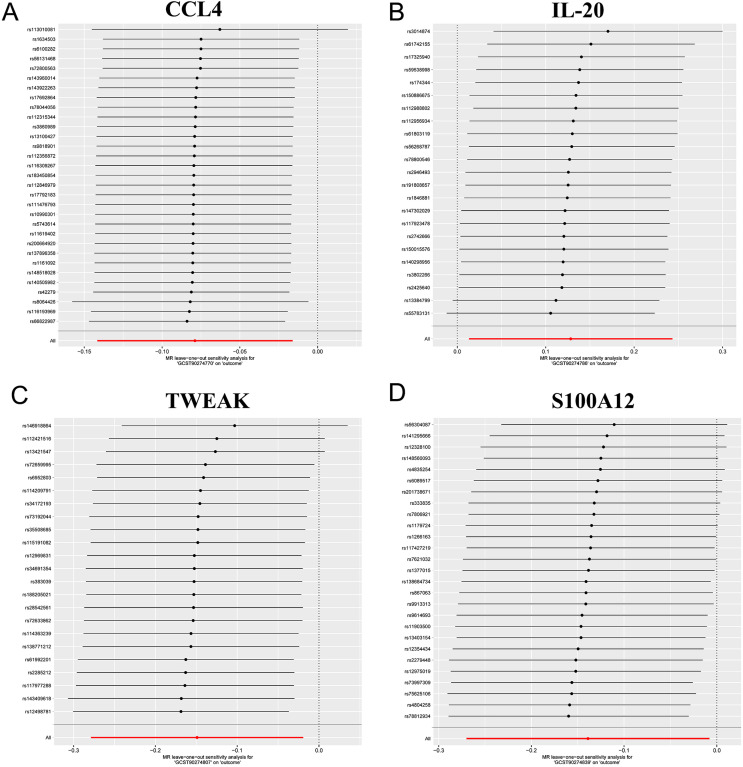
Leave-one-out sensitivity analysis plot for the effect of four circulating inflammatory proteins on TMDs. (A) CCL4, (B) IL-20, (C) TWEAK, (D) S100a12.

For the identified relationships, the F-statistic ranged from 19.55 to 982.2 for the IVs, indicating strong IVs and mitigating concerns over weak instrument bias. The Cochran’s Q tests, applying both MR-Egger and IVW methods, showed no significant heterogeneity for CCL4, IL-20, TWEAK, and S100A12 (all p-values >0.05) (Table S4). The absence of significant heterogeneity suggests that the effects of the genetic instruments are consistent, reinforcing the reliability of the observed causal links. This consistency supports the robustness of our findings as it indicates that the observed associations are unlikely to suffer the effect of varying genetic influences across different studies. Sensitivity analyses further validated these findings. Furthermore, analysis indicated no significant evidence of directional pleiotropy (Table S5), with intercepts and their standard errors (SE) being intercept =0.0036, SE=0.0083, p=0.6672; intercept = 0.0058, SE=0.0125, p=0.644; intercept =0.0312, SE=0.0188, p=0.1116; intercept =−0.0096, SE=0.0138, p=0.4908), respectively. This indicates that pleiotropy fails to significantly influence the causal estimates, reinforcing the robustness of our conclusions. Moreover, the leave-one-out analysis confirmed the stability of the results, showing that no single instrumental variable disproportionately impacted the overall findings. Additionally, leave-one-out analysis confirmed the lack of any single influential IV that could significantly skew the results if excluded (Figure 5).

## Discussion

This investigation marks the inaugural comprehensive MR study to examine the link between genetically determined levels of circulating inflammatory proteins and TMDs. However, several limitations deserve early consideration to provide context for interpreting the findings. First, although MR analysis reduces confounding factors and strengthens causal inference, it heavily relies on the strength of genetic instruments. Despite robust F-statistics indicating strong IVs, potential pleiotropic effects, and population-specific biases – given our reliance on data from European ancestry – may impact the generalizability of these results to broader and more diverse populations. Additionally, MR assumes that genetic variants affect the outcome only by exposure rather than via other pathways, an assumption that, if violated, could influence the interpretation of causal relationships.

By using the most extensive genome-wide association datasets currently available, our study discovered a potential link whereby elevated levels of S100A12 increase the risk of TMDs. Furthermore, we found compelling evidence from various MR methodologies suggesting that TMDs reduce levels of CCL4, IL-20, and TWEAK. The consistency of our findings, despite the different assumptions regarding horizontal pleiotropy, suggests that pleiotropic effects fail to fully account for the identified associations. Significantly, our research underscores the protective roles of CCL4, IL-20, and TWEAK against TMDs, aligning with and bolstering the case for anti-inflammatory treatments as viable preventive and therapeutic options for TMDs, in harmony with epidemiological evidence. Such insights into the dynamics between circulating inflammatory proteins and TMDs provide a foundational basis for exploring targeted anti-inflammatory therapies that could significantly impact the management of TMDs.^24^^,^^25^

Cytokines are pivotal in the processes of development, tissue remodeling, and the maintenance of homeostatic balance. They have been identified as key contributors in virtually all pathological conditions, ranging from cancer and cardiovascular diseases to specifically inflammatory conditions such as wound healing, infections, and autoimmune diseases.^26^^,^^27^ The role of cytokines in the pathogenesis of TMDs has been a subject of discussion for many years. Recent research has pinpointed the presence of inflammatory cytokines within the affected joint area or its surrounding environment, such as the synovial fluid, highlighting their significant involvement in TMDs.

CCL4, also recognized as macrophage inflammatory protein (MIP)-1β, plays a critical role in the chemotactic activities of the immune system. It activates the chemokine receptor CCR5, which participates in various immune responses. Secreted by CD8+ T cells, CCL4 can attract monocytes, natural killer cells, and other immune cells to sites of activity.^28^^,^^29^ Produced by monocytes, T/B lymphocytes, and neutrophils, CCL4 contributes to inflammation by attracting other leukocytes to the inflammation sites, leading either to the resolution of inflammation by macrophage-mediated efferocytosis or to the development of chronic inflammation.^30^ Evidence suggests that CCL4 may also promote tumor growth and progression by attracting regulatory T cells and pro-tumorigenic macrophages.^31^^,^^32^ It influences other cells within the tumor microenvironment, such as fibroblasts and endothelial cells, enhancing their pro-tumorigenic functions. Beyond its involvement in metabolic diseases and cancers, CCL4 plays a role in the development of systemic lupus erythematosus, multiple sclerosis, multiple myeloma, psoriasis, cystic fibrosis, and sarcoidosis. However, up to this point, the literature has no reported association between CCL4 and TMDs.^33^

IL-20 belongs to the IL-10 cytokine family and is notably expressed in various tissues that possess IL-20 receptors. This pro-inflammatory cytokine is involved in the pathogenesis of autoimmune diseases, notably psoriasis and rheumatoid arthritis, by its actions on keratinocytes and synovial fibroblasts. The administration of IL-20 can change the human epidermis akin to those seen in psoriasis by inhibiting the terminal differentiation of keratinocytes.^34^ Furthermore, IL-20 stimulates synovial fibroblasts to secrete pro-inflammatory molecules such as TNF-α, IL-1β, MMP-1, MMP-13, and MCP-1. This, in turn, attracts activated T cells, monocytes, dendritic cells, and neutrophils, damaging tissue. Beyond its link to rheumatoid arthritis by activating synovial fibroblasts, IL-20 also contributes to the development of nephritis by prompting kidney mesangial cells to produce various pro-inflammatory molecules, including MCP-1, RANTES, IP-10, and IL-6. However, reports on the involvement of IL-20 in TMDs remain scarce.^35^^-^^37^

TWEAK, belonging to the TNF ligand superfamily, engages in a range of biological roles by interacting with its receptor, fibroblast growth factor-inducible 14. Recent studies have shown that TWEAK triggers the production of inflammatory cytokines and plays roles in angiogenesis, apoptosis, and the healing and regeneration of tissues. It stimulates the release of various cytokines, including MMP-1, IL-6, IL-8, and RANTES, in synovial cells and fibroblasts, and produces intercellular adhesion molecule 1, E-selectin, IL-8, and MCP-1 in endothelial cells. Specifically, TWEAK promotes the production of matrix metalloproteinases in diseases affecting the joints, contributing to the deterioration of cartilage and bone by angiogenesis in synovial membranes and osteoclastogenesis.^38^^,^^39^ Additionally, it is implicated in hindering the differentiation of osteoblastic and cartilage cell precursors, disrupting natural repair processes. Nonetheless, the impact of TWEAK on the development of TMDs and its expression in joints in relation to TMDs severity remain unclear.

S100A12, part of the S100 protein family, consists of low-molecular-weight acidic proteins with specific cell-specific expression and calcium-binding motifs. Humans primarily produce and release it by neutrophil granulocytes.^40^^,^^41^ Upon activation by calcium, intracellular S100A12 interacts with various target proteins, influencing cell functions. Outside the cell, S100A12 shows cytokine-like properties as it undergoes significantly upregulation in inflamed tissues and heightened serum levels in individuals with a range of inflammatory, neurodegenerative, and cancerous conditions.^42^^,^^43^ Clinical data strongly support the utility of S100A12 as a precise and sensitive indicator to detect inflammation, with noted increases in its serum levels in those with rheumatoid arthritis.

The relationship between circulating inflammatory proteins and TMDs is complex and only partially understood, with current research providing inconsistent results on the specific roles of cytokines. The completion of the Human Genome Project has significantly advanced our understanding of disease-associated genes by functional genomics. In a similar vein, the literature has seen a marked increase in studies to find the genetic basis of susceptibility to TMDs by tools such as GWAS and focused examination of SNPs in areas that regulate or encode proteins. Functional genomics has heralded new opportunities for detailed investigation into the genetic factors contributing to TMDs, configuring a promising area for future research.

The results of this study may have substantial implications for future research and clinical practice. Understanding the causal roles of inflammatory proteins in TMDs offers new avenues for targeted therapies and risk prediction. Future studies should focus on validating these associations in diverse populations and exploring the therapeutic potential of modulating these proteins in clinical settings. Additionally, the identification of these proteins as biomarkers could inform early intervention strategies and help to develop personalized treatment approaches, ultimately improving patient outcomes.

### Limitations

Some limitations warrant consideration. Firstly, the influence of inflammatory cytokines on the functional outcomes of different TMDs subtypes remains unclear and necessitates further research. This is primarily due to a lack of GWAS data focusing on the functional prognosis of TMDs across these subtypes. Secondly, we adopted a p-value significance threshold of 5×10^-6^ for the GWAS data on exposure due to a scarcity of SNPs meeting the more stringent criterion of p < 5×10^-8^. This choice, while necessary, complicates the execution of further MR analysis and increases the potential for including less robust genetic instruments. Thirdly, most study participants were of European descent, which limits the generalizability of our findings to other ethnic groups. Ethnicity and comorbidities produce different results in these subgroups, which could significantly influence the applicability of our findings. Future studies should focus on validating these results across diverse populations to determine whether similar associations hold true across different ethnicities and clinical subgroups. Fourthly, while some associations reached statistical significance, it is important to differentiate between statistical significance and clinical relevance, particularly given the slight variations in inflammatory protein levels. Small effect sizes, although statistically significant, may fail to always translate into meaningful clinical impact, and further research is needed to understand how these findings could influence patient care. Fifthly, although MR minimizes the impact of confounders, it fails to eliminate the risk of residual bias. The method assumes no pleiotropic effects from the genetic instruments. However, undetected biases could still influence results, which must be recognized during interpretation. Sixthly, not all results from the MR-Egger, weighted median, simple mode, and weighted model analyses reached statistical significance in this investigation. However, given the significantly higher statistical power of the IVW method than the other techniques and the commitment of this study to a consistent direction in MR results, we consider the findings to hold substantial significance. Seventhly, our study primarily focused on inflammatory factors, which may fail to fully represent the diverse etiopathogenesis of different TMD subtypes. As different conditions and diagnoses have varying mechanisms, future research should aim to explore these differences and investigate distinct mechanisms for various TMD subtypes. Eighthly, existing research linking inflammatory proteins, such as CCL4, IL-20, TWEAK, and S100A12, with TMDs remains scarce. This lack of data limits our ability to draw definitive conclusions about the roles these proteins may play in TMD pathogenesis and progression. Moreover, without clinical data on mean pain duration and detailed TMD diagnoses (such as articular, muscular, or combined impairments), we are unable to fully explore the potential benefits of higher concentrations of these proteins for patients with chronic pain conditions and long pain duration. The impaired inhibitory modulation system in these individuals could also interfere with the outcomes but this aspect remains unexplored due to the lack of comprehensive clinical data.

## Conclusion

This study provides evidence of a potential relationship between inflammation and negative functional outcomes in TMDs, highlighting specific inflammatory proteins as contributing factors. Our findings suggest that reduced levels of CCL4, IL-20, and TWEAK may be associated with the deterioration in TMDs, whereas elevated levels of S100A12 might precede and potentially exacerbate adverse functional outcomes. These results reinforce the significance of inflammation in the pathogenesis of TMDs and suggest that targeting these cytokines could be a promising avenue for future therapeutic strategies.

The study contributes to the field by identifying specific inflammatory proteins that could serve as biomarkers or therapeutic targets for TMD management. By utilizing a comprehensive MR approach, our research advances understanding of the causal links between circulating inflammatory proteins and TMDs, offering novel insights that may inform both clinical and research directions.

Future studies should focus on validating these findings in larger and more diverse populations to determine the clinical utility of these cytokines as predictors of TMD outcomes. Additionally, exploring the mechanisms by which these proteins influence TMD progression and assessing their potential as targets in preventive strategies will be crucial for improving patient care.

### Ethics approval and consent to participate

This research was conducted using de-identified, publicly accessible summary data from earlier GWAS, thereby eliminating the need for ethical approval. The original GWAS from which these data were derived had already secured informed consent from all participants, ensuring the information was appropriately de-identified and made available for research use.
